# Esterified Lignin from Construction and Demolition Waste (CDW) as a Versatile Additive for Polylactic‐Acid (PLA) Composites—The Effect of Artificial Weathering on its Performance

**DOI:** 10.1002/gch2.202100137

**Published:** 2022-06-11

**Authors:** Ikenna Anugwom, Ville Lahtela, Mattias Hedenström, Samantha Kiljunen, Timo Kärki, Mari Kallioinen‐Mänttäri

**Affiliations:** ^1^ RE‐Source Platform LUT University Lappeenranta FI‐53851 Finland; ^2^ Fiber Composite Laboratory LUT University Lappeenranta FI‐53851 Finland; ^3^ SCI‐MAT Platform LUT University Lappeenranta FI‐53851 Finland; ^4^ Department of Chemistry Umeå University Umeå Sweden; ^5^ Center of Separation Technology LUT University Lappeenranta FI‐53851 Finland; ^6^ School of Engineering Science Department of Separation Science LUT University Lappeenranta FI‐53851 Finland

**Keywords:** composites, construction and demolition waste, coupling agents, deep eutectic solvents, lignin, versatile additives

## Abstract

Demand for sustainable packaging and building materials has increased the need for biobased additives. Biocomposites can often be exposed to different weather conditions and UV irradiation. Thus, additives to prevent the negative impact of weathering are generally added to composites. This study aims to evaluate using esterified lignin as an additive against weathering effects in polylactic‐acid (PLA) composites. Lignin is extracted from construction and demolition waste (CDW) wood using a deep eutectic solvent then esterified and tested as an additive in the fabrication of bio‐based composites. For comparison, lignin from birch is used as a raw material for an additive. Esterification is confirmed by solid‐state NMR analysis. Samples are exposed to artificial weathering for 700 hours and their impact strength and color change properties are measured. The results indicate that esterified lignin from CDW (CDW e‐lignin) as an additive protects the biocomposite from the weathering impact. The sample containing the CDW e‐lignin as an additive suffers only a 4.3% of reduction of impact strength, while the samples that contain commercial additives lose clearly more of their impact strength (from 23.1% to 61.1%). Based on the results CDW e‐lignin is a good additive to prevent weathering. As a conclusion, the esterified lignin from CDW, is a versatile additive for composite production.

## Graphical abstract

Valorization for construction and demolition waste wood extracted lignin into a multi‐purpose additive for the fabrication of polylactic‐acid‐based wood composite is reported.

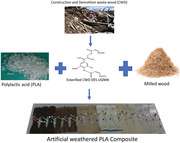

## 1 Introduction

Due to the continuous growth of the demand for sustainable solutions, the need for sustainable additives needed to produce bioplastics and bio‐composites can also be foreseen to increase in the near future. When the products should be biobased or biodegradable, it is not enough that one or two of the raw materials fulfill this criterion. This means that when moving towards biobased sustainable solutions, it is not enough to only have sustainable polymers and fillers but the additives and coupling agents also need to be sustainable. It has been forecast that bioplastics will have tremendous growth over the next decade, since additives are needed in their manufacturing; it can also be forecast that there will be a tremendous growth in the need for biobased additives.

Additives in plastic production help to improve their impact strength, chemical and heat resistance, and clarity of the produced plastic. They also help to increase weather resistance and provide color preservation properties to the produced plastics. In addition, they facilitate the processing of plastics. There are many types of polymer additives, for instance, plasticizers, stabilizers, fillers, flame‐retardants, and antioxidants and, thus, their market is large.^[^ ^1^ ^]^

In bio‐composites, at least one of the components must be derived from nature.^[^ ^1^ ^]^ A good example of a renewable biocomposite would be a wood plastic composite (WPC), which is fabricated from polylactic acid (PLA) and wood fibers. PLA is a biodegradable thermoplastic polyester derived primarily from renewable resources, via the fermentation of polysaccharides. PLA has several beneficial properties, such as biodegradability, easy processability, and good strength features. Therefore it has been widely studied for many applications in recent years, for instance in the manufacturing of packaging materials and construction materials.^[^ ^2^, ^3^, ^4^, ^5^, ^6^ ^]^ When considering the choice of PLA to be the plastic component in WPC, it is good to notice that PLA is not only produced from renewable feedstocks but requires four times less non‐renewable energy in its production than fossil‐based polymers.^[^ ^7^ ^]^ Despite the long list of positive advantages of PLA as a possible bioplastic material, PLA has some limitations as a material for composites, such as a narrow thermal processing window and high moisture sensitivity. Consequently, there are still some hurdles to overcome in terms of PLA as a biobased alternative plastic material, for instance, how to increase the lifespan of the bio‐composites made from PLA and wood fibers.

One of the factors causing reduction in the lifespan of WPC products used in outdoor environments is the exposure to UV radiation, which causes detrimental changes in the structures of WPC components.^[^ ^8^, ^9^ ^]^ Various filters, such as UV absorbers (UVA), hindered amine light stabilizers (HALS), and pigments, have been used to delay the onset of photo‐degradation resulting from exposure to UV radiation.^[^ ^8^, ^10^ ^]^ Lignin is the compound that absorbs UV irradiation in wood, thus, it would be an option to utilize lignin also as an additive to delay the onset of photo‐degradation resulting from the exposure of WPCs to UV radiation. In recent years, utilization of lignin as an additive has been studied with polyethylene (PE) and polypropylene polymers,^[^ ^11^, ^12^, ^13^, ^14^, ^15^ ^]^ but there are not many publications reporting on how lignin could be utilized as an additive in PLA based composites. However, there have been studies that have focused on the use of modified lignin as additives in WPC, and it has been reported that esterification of lignin used as additives enhanced the photostability of the WPCs produced from wood fibers and high‐density PE.^[^ ^11^, ^15^, ^16^ ^]^ Modification of lignin with maleic anhydride, as described in,^[^ ^17^ ^]^ resulted in esterification of lignin, and thus provided the possibility to use lignin as the additive to protect the PLA‐based composites from UV radiation‐induced photo‐degradation of the composite.

One of the attractive sources for lignin is construction and demolition waste (CDW) wood. It is available in great volumes, for example, 36% of all the waste generated in the EU results from construction and demolition.^[^ ^18^ ^]^ In Finland, CDW includes 36% of wood material, which means that the waste is a good source of lignin.^[^ ^19^ ^]^ Furthermore, having a process that enables for us the recovery of lignin from the CDW wood with the novel green solvents and the possibility to modify the new type of lignin, is beneficial from an environmentally friendly point of view. Additionally, their utilization as an additive for the fabrication of WPC motivated our previous research. In our earlier studies, we showed that the CDW originating lignin, which was modified, functioned well as a coupling agent in the production of PLA‐based WPC.^[^ ^17^ ^]^ Herein, the aim of the current work is to test if the modified DES‐lignin can also introduce antioxidant properties to the produced composite. Therefore, modified deep eutectic solvent‐lignin can act as a multifunctional agent, which can preserve the PLA‐based biocomposite from weathering.

## 2 Experimental Section

### 2.1 Materials

Six different composites were studied: three included commercial antioxidants and a commercial coupling agent, two included modified lignin materials, and one was made without antioxidant as a reference. The reference composite was made from PLA polymer (PLA 3051D), cellulose fiber (Arbocel B 800), a coupling agent (Fusabond E226), and a lubricant (Struktol TPW 113). Three of the produced biocomposite samples contained commercial additives to protect from weathering (Tinuvin 770F, Irganox 1010, Chimassorb 81), whereas the composites, which contained lignin, had as the weathering protecting additive only the modified lignin. (See **Table** **1**). Lubricant was included in all the composites. Lignin acted also as the coupling agent in the biocomposite samples, in which it was tested as the additive to prevent the influence of weathering. An earlier study presented a successful result where modified DES‐lignin functioned as a coupling agent in PLA‐based WPC, therefore, in this current study,^[^ ^17^ ^]^ samples that contain modified DES‐lignin in their formulation did not need the addition of a commercial coupling agent. Furthermore, the hypothesis herein was that modified DES‐lignin can act as a coupling agent as well as an antioxidant (a multifunctional additive), therefore, the other composite prepared, their formulation included commercial antioxidant as well as coupling agent.

**Table 1:** The composition of the composites manufactured and studied here. Amounts of shares are given in percent (%) based on the gravimeter

					Antioxidant^c)^				
Composite	PLA	Fiber	C.a.^a)^	Lubr.^b)^	*1*	*2*	*3*	*4*	*5*
Reference	86	10	3	1	–	–	–	–	–
PLA/A‐o.1	83	10	3	1	3	–	–	–	–
PLA/A‐o.2	83	10	3	1	‐	3	–	–	–
PLA/A‐o.3	83	10	3	1	‐	‐	3	–	–
PLA/e‐DESL‐CDW	86	10	–	1	–	–	–	3	–
PLA/e‐DESL	86	10	–	1	–	–	–	–	3

^a)^ Coupling agent

^b)^ Lubricant

^c)^ *1*) Tinuvin 770F; *2*) Irganox 1010; *3*) Chimassorb 81; *4*) Esterified lignin from CDW; *5*) Esterified lignin from native birch.

The matrix in the composite was commercially available PLA (polylactide) polymer supplied by NatureWorks LLC with the trade name PLA 3051D. The product was delivered as crystalline pellets, and the specific gravity of the PLA 3051D was 1.25 g cm^−3^ and the melt flow index was 10–25 g/10 min (at 210 °C/2.16 kg). The glass transition temperature and crystalline melt temperature of this PLA resin are between 55 and 65 °C and 150 and 165 °C, respectively. PLA 3051D was especially designed for injection molding applications. Arbocel B 800, supplied by J. RETTENMAIER & SÖHNE GmbH + Co KG, was used as fibers in the composites. The cellulose content of Arbocel B 800 was approximately 99.5% with a specific gravity of 1.50 g cm^−3^. The average fiber length and thickness were 130 and 20 µm, respectively. The coupling agent in the reference and commercial antioxidant composites was anhydride‐modified PE (MAPE), Fusabond E226 (DuPont). The density of MAPE was 0.93 g cm^−3^ with the melt flow index of 1.75 g/10 min (at 210 °C/2.16 kg). Struktol TPW 113, in a pellet or powder form with a specific gravity of 1.005 g cm^−3^, was used as the lubricant agent in all studied composites.

Three commercial antioxidant additives (absorber, stabilizer, antioxidant) were tested in this study: Tinuvin 770, Irganox 1010, and Chimassorb 81. They were obtained from BASF Scheweiz AG. The selection of additives was based on their dissimilar action as a part of structure in wood polymer composites. Tinuvin 770 (Bis(2,2,6,6,‐tetramethyl‐4‐piperidyl)sebaceate), which was a low molecular weight HALS, was delivered as a white crystalline granule and the melting range was 81–85 °C. Irganox 1010 (Pentaerythritol tetrakis(3‐(3,5‐di‐tert‐butyl‐4‐hydroxyphenyl)propionate)), which was a sterically hindered phenolic primary antioxidant, was delivered as a white free‐flowing powder and the melting range was 110–125 °C. Chimassorb 81 ([2‐hydroxy‐4‐(octyloxy) phenyl]phenyl), an ultraviolet light absorber (UVA), was delivered as a slightly yellow powder and the melting range was 47–49 °C.

In addition to the commercial additives, lignin‐based materials from two different sources (native birch wood and CDW that was based mainly on softwood) were used as additives in the composites. Lignin extraction from both native and CDW wood has been described in previous publications^[^ ^20^, ^21^ ^]^ and the lignin recovered from wood with DES has here been called “DES‐lignin”. Briefly, DES solution (1:9 choline chloride: lactic acid) was used as the solvent for lignin extraction. Firstly, 10% wood loading in DES solution was treated at 105 °C in an oil bath for 18 h. Subsequently, the mixture was thoroughly washed with a mixture of ethanol and water (2:1, v/v). After that, the mixture was filtrated through a filter paper in a ceramic Büchner funnel under vacuum. All the solutions from the washing of the undissolved wood were collected, and ethanol was then evaporated using a rotary evaporator at 60 °C. Deionized water was added to the concentrate to induce the precipitation of lignin. Finally, the lignin was separated with a centrifuge. The recovered lignin was freeze‐dried. The DES‐lignin was esterified with maleic anhydride, following the procedure described in earlier publications.^[^ ^22^, ^23^ ^]^ A detailed description of the procedure for esterification of the DES‐lignin has been reported and discussed in an earlier publication.^[^ ^17^ ^]^

### 2.2 Producing the Biocomposite Samples

The biocomposite samples for testing were produced in a Boy 30 injection‐molding machine. Due to the weak compatibility of fibers within the other components in the matrix, the injection process was performed twice. Achieving the better adhesion for matrix and fibers, materials were compounded twice in the production, which cannot be applied in the industrial processing. Although, double pass through the injection barrel could be regarded as a practical and smart idea for a scientific work. But this method cannot be applied in industrial manufacturing processes due to cost. First, the materials were extruded through the apparatus without a molding, and then crushed with a Shini SG‐1635N low‐speed granulator (Shini Plastic Technologies, Inc.), equipped with a 5.00 mm sieve. The crushed materials were re‐prepared with the injection‐molding machine. The used parameters in the injection molding were the following: melting temperature 160–180 °C, injection pressure 8.0–9.1 MPa, and injection time 3–5 s. The mold of injection molding produced the finished specimens for testing.

### 2.3 Testing and Analysis of the Modified Lignin and the Biocomposite Samples

#### 2.3.1 Solid‐State ^13^C NMR Spectroscopy

Solid‐state ^13^C cross‐polarization magic angle spinning (CP‐MAS) experiments were acquired on a Bruker 500 MHz Avance III spectrometer operating at a ^13^C frequency of 125.75 MHz, equipped with a 4 mm MAS probe. All samples were analyzed as dry powders (approximately 80 mg) packed into 4 mm ZrO_2_ rotors. A CP‐MAS pulse sequence with a 1 ms contact pulse was used with ramped amplitude on the ^1^H pulse (50–100%). Spinal64 ^1^H decoupling was applied during the 23 ms acquisition time. 8192 scans were accumulated for each spectrum and the spin‐rate was 10 kHz. Adamantane was used as an external chemical shift reference. All experiments were acquired at ambient temperature and processed in Topspin 3.2 (Bruker Biospin, Germany).

#### 2.3.2 Artificial Weathering Performance

The artificial weathering test included accelerated intervals of UV light and water spraying test in a test chamber (Q‐Sun Xe‐3 Xenon Test Chamber) according to standard EN ISO 4892‐2. The influence of UV light (340 nm) and water spray was analyzed by measurement of surface color of the stressed specimens, after 25, 50, and 100 h, following measurements after every 100 h until the exposure of 700 h. The color of the specimens was measured with a Minolta CM‐2500d spectrophotometer (Konica Minolta Sensing Inc., Japan) with the following settings: illumination D65, observer 10°, and illumination area 8.00 mm. The specular component included (SCI) and specular component excluded (SCE) influence of gloss was measured simultaneously. The CIELAB color system was used to measure the colors in *L*, *a*, and *b* coordinates, wherein *L* represents the lightness coordinate from 100 (*white*) to 0 (*dark*); *a* represents the red (*+a*) to green (−*a*); and *b* represents the yellow (*+b*) to blue (−*b*) coordinate. The color alteration was calculated according to the following equation:

(1) $$\begin{array}{c} \Delta E=\sqrt{{\left(\Delta L\right)}^{2}+{\left(\Delta a\right)}^{2}+{\left(\Delta b\right)}^{2}} \end{array}$$

Where: Δ*L*, Δ*a*, and Δ*b* represent the differences between the initial and measured values of *L*, *a*, and *b*, respectively. The color for three replicates was measured at three locations on each sample.

The Charpy impact strength for unnotched samples tested in a flatwise position was determined with a Zwick 5102 Model impact tester based on the EN ISO 179‐1 method. Three replicates with the dimension L × W × H: 50 mm × 6 mm × 4 mm were tested, due to the limited amount of available material, mechanical test included same replicant amount with the weathering test. Before the test, the samples were conditioned at 23 ± 1 °C and 50% relative humidity for several days.

Surface morphology of the samples and the cross‐sections of the impact strength samples were studied with a scanning electron microscope (SEM) apparatus, Hitachi SU3500 (Chiyoda, Tokyo, Japan) with the following operation conditions: voltage 15.0 kV, vacuum 90–100 Pa, and magnification varying between 50 and 500. Surfaces were observed directly after processing and after the artificial weathering exposure. In addition, the break‐surfaces of the tested impact strength samples were studied with an SEM.

### 2.4 Contact Angle (CA) Measurements

The CA of the samples was measured by using a KSV CAM 101 instrument (KSV Instruments Ltd., Finland) connected to a CCD camera (DMK 21F04, The Imaging Source Europe GmbH, Bremen, Germany). The measurements were done to determine the static CAs on the surface of the fabricated composite before and after artificial weathering with the sessile drop method. The volume of the droplet of DI water, which was dropped on the membrane surface with the aid of a micro syringe, was about 5 µL. The measurements were done at room temperature. The reliability of the measurements was ensured by taken values of at least ten independent points for each sample. The average of recorded data was considered as the final CA. The captured images were treated by curve fitting analysis with CAM 2008 software to determine the CA.

### 2.5 Fourier Transform Infrared Spectroscopy

In this work, Fourier transform infrared spectroscopy (FTIR) was performed to evaluate surface chemistry changes of the fabricated composites before and after the artificial weathering exposure. The FTIR spectra were recorded using a Perkin Elmer Frontier spectrometer with a universal ATR module (Diamond crystal). FTIR spectra of composite samples were taken in the 4000–400 cm^−1^ wavenumber range with the resolution of 4 cm^−1^. All the spectra were the acquisition of four scans with the data interval of 1 cm^−1^ at the absorbance mode. At the final step, the co‐added spectra were processed by ATR correction, then baseline correction, and finally normalized. The peaks were analyzed without smoothing the data. The data was presented in the form of indices and concentrations of the functional groups of interest.

## 3 Results and Discussion

### 3.1 ^13^C NMR Spectra of Unmodified, Modified, and Fabricated WPC

**Figure 1:**
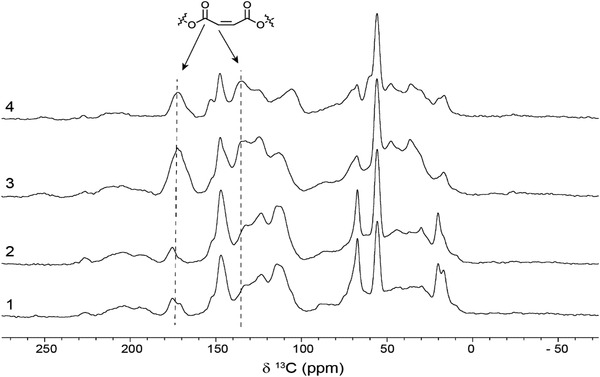
^13^C NMR spectra of DES‐lignin from 1) birch wood, 2) non‐esterified CDW waste wood, 3) esterified birch wood, and 4) esterified CDW waste wood.

**Figure 2:**
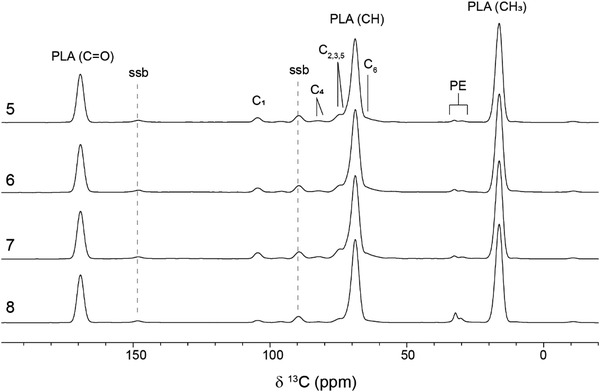
^13^C NMR spectra from 5) PLA (polylactic acid) with cellulose and esterified DES‐Lignin (birch) as coupling agent, 6) PLA (polylactic acid) with cellulose and esterified DES‐CDW Lignin as coupling agent and 7) PLA (polylactic acid) with cellulose and esterified DES‐Lignin as coupling agent 8) PLA (polylactic acid) with cellulose and commercial coupling agent. Ssb = spinning sidebands. All samples included in the NMR analysis are without any commercial antioxidant.

NMR spectra of the unmodified (spectrum 1 and 2) and esterified lignins (spectrum 3 and 4) used in this study are shown in **Figure** **1**. A significant increase in peak intensities of the carbonyl peak at 172 ppm and the unsaturated carbon at 135 ppm from the maleate functionalization is observed in the esterified lignins, indicating successful esterification of the extracted lignin. Spectra of the produced biocomposites including either commercial coupling agent or esterified lignin are presented in **Figure** **2**. The dominant features of the spectra are the three peaks from PLA located at 169.5 ppm (carbonyl), 68.9 ppm (CH), and 16.4 ppm (CH_3_).^[^ ^24^ ^]^ In addition, cellulose peaks were observed at 105.3 ppm for C1, 89.30 ppm, and 84.0 ppm for C4 of crystalline and amorphous cellulose respectively, 75.0 and 72.6 ppm for C2, C3, and C5, and 63.04 ppm for C6.^[^ ^25^ ^]^ Signals from lignin cannot be seen from the spectra of the biocomposite samples due to the strong overlapping with the PLA and cellulose originating peaks. The resonance peaks attributed to the PE component, which was used as coupling agent in the composites, were observed at the chemical shifts of 33.0 and 30.5 ppm, assigned to crystalline and amorphous methylene groups, respectively.^[^ ^26^ ^]^

### 3.2 Weathering Performance

**Figure 3:**
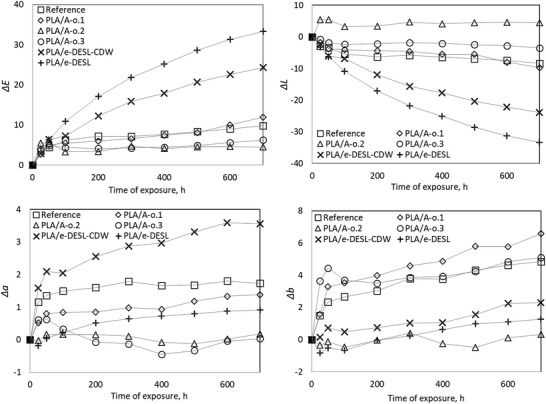
Total color alteration (Δ*E*), and alterations of lightness (Δ*L*), red/green component (Δ*a*), and yellow/blue component (Δ*b*) as a function of exposure time for the studied materials.

Weathering performance is presented as a point chart in **Figure** **3**, where exposing time is presented in the x‐axis. As already presented earlier, the SCI and SCE influence on gloss were measured simultaneously. Comparison between the SCI and the SCE measurements did not show any significant differences between each other, and the results presented in Figure 3. are from the SCI measurements only.

Comparison of the values indicating “yellowing” (Δ*b* in Figure 3) of the samples shows that the esterified lignin has prevented the yellowing due to the weathering exposure in the experiments more efficiently than the commercial weathering preventive additives. The esterified lignin, which originates from native birch, has also prevented the color change in the coordinates from red to green well ((Δ*a*) component in Figure 3) and maintains the color change on this coordinate at the same low levels as the commercial additives. However, compared to those, the esterified lignin originating from CDW has not had as protective an impact on the composite color measured as the change from red to green. The difference in the results between the two different lignin compounds could be explained by the differences in their chemical structures. Softwood lignin (in CDW) includes 95% of coniferyl alcohol and 5% of p‐coumaryl alcohol (C_10_H_9_O_2_) while hardwood lignin consists of coniferyl alcohol (C_10_H_12_O_3_) and sinapyl alcohol (C_11_H_14_O_4_). It has been found that under weathering exposure, softwood lignin degrades faster compared to the hardwood lignin.^[^ ^27^ ^]^

**Figure 4:**
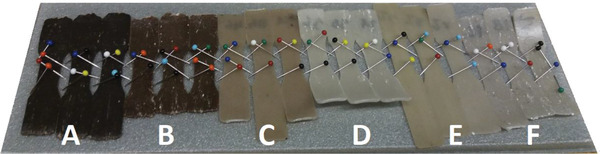
Images of the studied samples (A = PLA/e‐DESL, B = PLA/e‐DESL‐CDW, C = PLA/A‐o.1, D = PLA/A‐o.2, E = PLA/A‐o.1, F = Reference, names based on the information of Table 1) before artificial weathering exposure.

The lightness value (Δ*L* in Figure 3) of material dominates thus also here the behavior of weathering performance. Materials that are initially darker have naturally a higher potential and a greater ability for color change. When lignin is used as the additive in biocomposites, it gives the composite a very dark color, see **Figure** **4**. Thus, it was expected that in comparison to the lightness values the change due to the weathering is the biggest for the samples in which lignin additive has been used. In order to minimize this effect, new lightness change values, where actual color change (Δ*L*) is compared to the total potential of color change (*L*:100—initial:*L*) during the test, were calculated (See **Table** **2**).

**Table 2:** The relative lightness changes. Values are given in percent (%) based on the actual color change (absolute value of color change compared to initial condition) in proportion to the available potential maximum color change (100—initial value of Δ*L*)

Composite	25 h	50 h	100 h	200 h	300 h	400 h	500 h	600 h	700 h
Reference	7.7	9.1	14.3	16.6	15.2	16.9	18.1	19.6	21.9
PLA/A‐o.1	4.7	9.6	10.3	11.2	11.7	14.0	13.9	20.3	24.6
PLA/A‐o.2	18.0	17.9	10.8	11.2	15.4	13.5	14.9	15.2	14.9
PLA/A‐o.3	1.6	4.1	5.1	4.8	4.0	4.5	5.3	6.0	7.7
PLA/e‐DESL‐CDW	3.5	8.9	10.3	17.8	23.2	26.2	30.3	33.0	35.6
PLA/e‐DESL	4.4	8.6	14.8	23.3	29.6	34.13	38.9	42.4	45.3

The relative lightness changes (Table 2) correspond to the changes in actual colors (Figure 3) and show that the commercial antioxidants had the best ability to restrict lightness changes during the weathering stress. It can be supposed that the use of only minor amounts of wood fibers in these biocomposites contributes to the composite samples not suffering from big changes in the lightness values.^[^ ^28^ ^]^ However, as already said, lignin gives the composite a darker color and it is as compound sensitive for degradation under UV irradiation (see Figure 4.). This resulted, as expected, in a bigger change in the lightness values in the composites, which contained lignin as an additive compared to the other tested samples.

**Figure 5:**
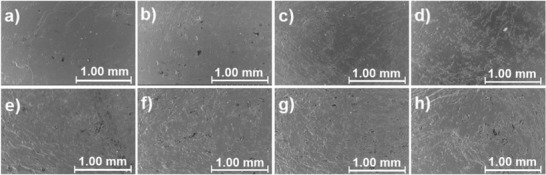
SEM figures from the surface of samples a–d) before and e–h) after exposure of artificial weathering at the magnification of 50×. Images from reference (images: a,e), least color changed (PLA/A‐o.2) (images: b,f), and the recycled lignin (PLA/e‐DESL‐CDW) (images: c,g) and native lignin (PLA/e‐DESL) (images: d,h) materials

The influence of artificial weathering on the surfaces of the composite samples is presented in **Figure** **5**, which shows SEM images from the surfaces of samples. As expected, exposure to weathering caused damage on the sample surfaces. For example, a very clear difference between the non‐weathered reference sample (Figure 5a) and weathered reference sample can be observed. Unfortunately, the SEM analysis of the sample surfaces did not reveal a clear difference between the weathering resistance of the samples containing the commercial additives and the samples that contained the esterified lignin as an additive.

### 3.3 Changes in Chemical Composition Due to Weathering

**Figure 6:**
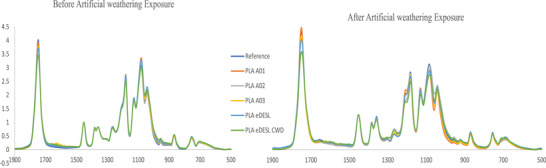
FTIR spectra of the composite A) before exposure to artificial weathering and B) after the artificial weathering exposure, the spectra show the drop in the signal at 1745 cm^−1^.

The changes in chemical structure of the composites due to the weathering were examined through measuring the FTIR spectra before and after the weathering exposure. The typical changes to PLA due to photo oxidation by UV irradiation often lead to the formation of carbonyl products, which are identifiable in IR as anhydride functions and are characterized by the formation of new bands at 1845 cm^−1^.^[^ ^29^, ^30^ ^]^ However, this was not the case in this work; there were no signals identified at 1845 cm^−1^ for the IR spectra before and after exposure to the artificial weathering (**Figure** **6**). Only a marked reduction of IR signal band between 1745–1754 cm^−1^ for all composite samples after the artificial weathering exposure, Figure 6. It should be noted that the composites containing modified lignin as additives and the ones containing commercial additives show a similar trend in decline, which could be because both the commercial and lignin‐based additives contain similar chromophoric groups that are responsible for the lower chain scissions occurring in the macromolecular chain. It is known that a combination of polymer and natural fibers, such as wood, increases the amount of oxygen in the composite structure, thus, there is carbonyl functionality, which in turn makes the composites more susceptible to degradation.^[^ ^31^ ^]^ The wood fibers tend to degrade due to the exposure to UV irradiation, and this involves a photo‐induced breakdown of lignin, generating additional chromophoric groups such as carbonyls, carboxylic acids, quinines, and hydroperoxy radicals.^[^ ^32^ ^]^ This could be attributed to lower chain scissions occurring in the macromolecular chain because of the inclusions of additives.^[^ ^16^, ^33^, ^34^, ^35^, ^36^ ^]^

### 3.4 Changes in Mechanical Performance Due to the Weathering

**Figure 7:**
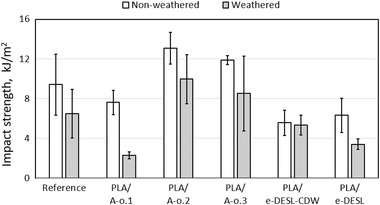
Impact strength properties of the studied materials before weathering (white column) and after weathering (gray column). The error bars describe standard deviations.

The impact strength properties of the studied biocomposites are shown as a clustered column in **Figure** **7**. Generally, impact strength decreased after the exposure to artificial weathering. For example, a reduction of 31.1% was seen for the reference composite. The reductions in the impact strength of the samples, which included commercial antioxidant (PLA/A‐o.2) and absorber (PLA/A‐o.3) were at a similar level: 23.8% and 28.1%, respectively. Composition and mixtures of recipes influence the durability of material. Typically, phenolic antioxidant and HALS have demonstrated the same stabilization efficacy separately, but in combination have caused faster and a higher amount of the degradation products.^[^ ^37^ ^]^ Treatment parameters might have a significant effect on the results because, for example, HALS loses its activity at temperatures above 120 °C.^[^ ^38^ ^]^ Thus, WPC having HALS degrades faster compared to the pure polymers, due to the hydroscopic properties, since water in the material operates as a catalyst for oxidation. Furthermore, WPC material might also include metal particles which might also play a role in catalyzing the oxidation process.^[^ ^28^ ^]^ In this study, the material that included HALS stabilizer lost a remarkable share of impact strength in weathering exposure, reduction was 61.1% (PLA/A‐o.1). However, with the material that included modified CDW DES‐lignin (PLA/e‐DESL‐CDW), impact strength was reduced by only 4.3% while with the esterified DES‐lignin from birch (PLA/e‐DESL), the reduction was 46.1%, see Figure 7. The results thus show that the use of esterified CDW DES‐lignin (PLA/e‐DESL‐CDW) in the structure of composites enabled retaining of mechanical properties despite the artificial weathering.

It has been reported earlier that exposing WPCs to UV radiation degrades hydrophobic lignin and leaves hydrophilic cellulose at the surface. This increases surface wettability and causes the surface to become more sensitive to moisture, which then can lead to the loss in strength of the WPC. However, the hygroscopicity of wood can be reduced by replacing some of the hydroxyl groups with alternative chemical groups. By acetylation, the moisture performance of wood composites can be improved. Acetic anhydride reacts with hydroxyl groups in the wood cell wall to yield and acetylated fiber.^[^ ^35^, ^39^, ^40^ ^]^ The use of CDW DES‐lignin and the esterified DES‐lignin from birch as an additive resulted in a clear increase in the CA of the composite sample meaning that the composite samples were more hydrophobic than the reference sample or those samples that included the commercial additives (See **Table** **3**). Thus, they might have attributed to the improved moisture resistance of the composite samples. The artificial weathering caused, however, a clear decrease in hydrophobicity of the composite samples that contained lignin as an additive.

**Table 3:** The CA values of the fabricated composite before and after artificial weathering exposure

Contact angle	Before artificial weathering	After artificial weathering	CA difference
Ref_1	82.5 ± 1.3	74.2 ± 1.4	8.3
PLA_AO1	69 ± 1.1	81 ± 4.2	−12
PLA _AO2	81.7 ± 0.8	81.6 ± 1.8	0.1
PLA _AO3	82.6 ± 0.9	81.5 ± 3	1.1
PLA_e_DESL_CDW	85.3 ± 1.5	71.2±2.4	14.1
PLA_e_DESL_	88.7 ± 4.2	67.3 ± 1.2	21.4

The reason why only the use of esterified CDW DES‐lignin (PLA/e‐DESL‐CDW) in the structure of composites enabled better retaining of mechanical properties despite the artificial weathering compared to the esterified lignin from the birch might originate from the differences in lignin structures. Typically, there is a reduction in glass transition temperatures (*T*_g_) of lignin samples modified by esterification, because in the esterification the removal of hydroxyl groups decreases the inter‐ and intra‐hydrogen bonding capability of lignin. As a result, the weaker intermolecular bonds allow more mobility of lignin macromolecules at elevated temperatures.^[^ ^41^ ^]^ Haw et al. measured relative intensities of two signals in heat‐treated and untreated wood spectra to study the degree to which β‐O‐4 linkages in lignin are cleaved during the thermal modification.^[^ ^42^, ^43^ ^]^ According to this, the majority of the β‐O‐4 linkages in hardwoods’ syringyl units were mostly cleaved. This extensive aryl‐ether bond cleavage is probably due to moisture and heat during the artificial exposure process.^[^ ^42^, ^43^, ^44^ ^]^ Furthermore, thermal treatment in the presence of moisture partially depolymerizes wood lignin by hydrolyzing the aryl ether linkages of syringyl and guaiacyl units, which can then lead to the formation of free hydroxyl phenolic groups and α‐ and β‐carbonyl groups. Moreover, during thermal treatment of modified lignin there are suggestions that guaiacyl units are linked by carbon‐carbon bonds and hence the content of condensed guaiacyl structures tends to increase.^[^ ^45^ ^]^ Through the formation of a condensation which is typically notice when a carbon–carbon bonds is formed, this indicates that only guaiacyl units are condensed during thermal treatment. These bonds cannot be formed between syringyl units because of the presence of a methoxyl group at positions where the carbon‐carbon bond is responsible for the condensation reaction formation.^[^ ^46^ ^]^ It was reported that lignin condensation probably improves more the strength of thermally modified softwoods compared to hardwoods, since the most notable differences between hard and softwoods are principally the fact that softwood lignin contains mainly guaiacyl unit, while hardwood lignin contains both guaiacyl and syringyl units. Furthermore, the cleavage of the β‐O‐4 bonds during thermal treatments such as artificial weathering exposure is more extensive in hardwoods than in softwoods. Hardwood syringyl lignin is reported to depolymerize to a greater extent than softwood guaicyl lignin when exposed to steam treatment.^[^ ^42^, ^43^, ^44^, ^46^ ^]^

### 3.5 Change in the Hydrophilicity Due to the Weathering

The CAs of the composite were taken before and after artificial weathering exposure, to compare the durability of the different additives including the modified lignin compounds as an additive in the fabricated PLA wood composite towards light‐induced degradation. Before the artificial weathering, all the samples exhibited a hydrophobic characteristic since their CAs were between 62–85° (presented in Table 3). This result is consistent with those found in the literature.^[^ ^33^, ^47^ ^]^ Further, blends formed by modified lignins showed high CA values, indicating a slight improvement in the hydrophobic character of PLA. After artificial weathering exposure, the samples with antioxidant agent showed slight reductions in their CA, less than 2°, indicating their high tolerance to the weathering, while the composites that had as additives modified lignins exhibited a 14° (CDW DES‐lignin) and 21° (Birch DES‐lignin) reduction in the CA, respectively.

## 4 Conclusions

Esterified lignins, which were prepared via extraction from CDW wood or from native birch using DES as a solvent, were tested as additives to prevent weathering performance of PLA composites. The esterification of the used lignin could be confirmed from the NMR spectra. The results showed that the esterified DES‐lignin, which was originating from CDW, acted as an additive to enable retaining of the impact strength properties of the biocomposite sample. The difference compared to the reference sample, which did not contain any antioxidative additive, was very clear: impact strength reduction of the reference sample was 31.1%, while only 4.3% impact strength reduction was recorded for the sample in which CDW DES‐lignin was utilized as an additive. The reduction of the impact strength of the composite samples containing commercial additives, which are usually added to prevent the influence of weathering, were 23.8%, 28.1%, and 61.1%, respectively. Thus, having modified lignin as an additive provides the composite enhanced impacted strength. However, it must be noted that utilizing the esterified lignin as the additive in biocomposites gives a dark, brownish color to the composite and makes it prone to fading of the color due to exposure to UV irradiation. In any case, the results of this study and our earlier study.^[^ ^17^ ^]^ together demonstrate that esterified lignin that is originating from CDW is a promising versatile additive for composite production. This means that lignin recovered from CDW could via a simple esterification process be refined to value‐added biobased products.

## Conflict of Interest

The authors declare no conflict of interest.

## Data Availability

The data that support the findings of this study are available from the corresponding author upon reasonable request.
